# Could the Combined Administration of Bone Antiresorptive Drug, Taxanes, and Corticosteroids Worsen Medication Related Osteonecrosis of the Jaws in Cancer Patients?

**DOI:** 10.1155/2018/4021952

**Published:** 2018-05-29

**Authors:** Giacomo Oteri, Giuseppina Campisi, Vera Panzarella, Ilaria Morreale, Riccardo Nucera, Olga Di Fede, Antonio Picone, Antonia Marcianò

**Affiliations:** ^1^Unit of Dentistry, AOU Policlinico “G. Martino”, Messina, Italy; ^2^Department of Biomedical and Dental Sciences and Morphofunctional Imaging, University of Messina, Messina, Italy; ^3^Department of Surgical, Oncological and Oral Sciences, University of Palermo, Palermo, Italy; ^4^Clinical Pharmacology Unit, Sicilian Regional Center of Pharmacovigilance, Azienda Ospedaliera Universitaria Policlinico P. Giaccone, University of Palermo, Palermo, Italy; ^5^Humanitas Centro Catanese di Oncologia, Catania, Italy

## Abstract

The study presents a report of 58 metastatic cancer patients who developed osteonecrosis of the jaws after being treated with zoledronic acid and taxanes, plus corticosteroids. A retrospective analysis of data registered in the archives of two Italian osteonecrosis of the jaws treatment centers, who are based at the University of Messina and at the University of Palermo, was performed in order to study, in these patients, demographic data and characteristics such as frequency of cancer location, lines of therapy, frequency of cancer drugs, presence/absence of oral trigger, number, location, and stage of jaw osteonecrosis. It was found that the majority of patients developed advanced stages of osteonecrosis, frequently complicated with infection. It was hypothesized that the concurrent administration of chemotherapeutic agents could be eventually considered as a factor able to allow a faster worsening of the clinical manifestation through the exacerbation of soft tissue defects, due to chemotherapy drugs.

## 1. Introduction

Medication related osteonecrosis of the jaws (MR-ONJ) constitutes an important issue in the field of oral medicine and surgery as it is regularly encountered in oral pathology services. The majority of MR-ONJ cases are associated with the high-risk cancer patients [1, 2]. Osteonecrosis of the jaws in patients undergoing treatment with antiresorptive and/or antiangiogenic agents appears to be caused by the combination of a vascular supply deficit and a remodeling and bone regeneration deficit [3]. Bisphosphonates (BPs), denosumab but not immunosuppressive or chemotherapy drugs, are the major cause of MR-ONJ [4–6]. Nevertheless it has been reported that the simultaneous administration of chemotherapy drugs increases the severity of the disease, especially the incidence of soft tissue defects [7].

The capacity of antineoplastic drugs to accelerate MR-ONJ is likely attributed to their ability to block endothelial cell migration and proliferation [8, 9]. In a study by Bi et al. mice treated with bisphosphonates and dexamethasone all developed osteonecrosis. In this in vivo experimental model the authors found that administration of zoledronic acid (ZA), dexamethasone, and docetaxel leads to bone necrosis and intense inflammation within the extracted alveolar socket well beyond the period of natural wound healing. The authors found that injections of zoledronate, dexamethasone, and docetaxel developed significantly larger areas of dead bone at both 3 and 12 weeks when compared with mice treated with zoledronate and dexamethasone [7].

MR-ONJ is strongly related to the long-term use of bisphosphonates and mechanical trauma [10]. Recent retrospective analyses of human MR-ONJ cases demonstrate a correlation among the use of chemotherapy drugs and immunosuppressive agents and the development of MR-ONJ [7].

Bisphosphonates and denosumab are drugs widely used on a worldwide scale with clear benefits for oncological patients that have been clinically proven. They result in a quality of life improvement for oncological patients with bone metastases but, on the other hand, the appearance of osteonecrosis of the jaws dramatically affects general health and quality of life of patients suffering of this complication with high morbidity. Quality of life values significantly decrease with worsening MR-ONJ [11–13]. To hold in consideration the oral health quality of life (OHQoL) is recommended by The WHO to assess the subjective and objective functional status of an individual's oral health and the impact of oral complications on daily living and QoL [14].

To pay greater attention to predisposing risk factors translates into better prevention strategies. Therefore the objective of this study was to report about the observation of cancer patients treated with taxanes, bisphosphonate, and denosumab plus corticosteroids who developed osteonecrosis of the jaws.

## 2. Methods and Materials

The study protocol was approved in date 03.27.2017 by the Ethical Committee of the Academic Hospital of Messina (n. 38/17). The files of the two Italian osteonecrosis of the jaws treatment centers, who are based at the University of Messina and at the University of Palermo, served as a source of material for this study. Files were systematically searched for cases of MR-ONJ in metastatic cancer patients treated with taxanes during a 10-year period (2006–2016). Cancer patients prior to start, undergoing or with a history of previous treatment with bisphosphonates or denosumab for the treatment of bone metastases, are regularly referred to the dental specialists at these MR-ONJ treatment centers, by the chief oncologist and/or primary care physician.

As part of the clinical care routine medical anamnesis is collected in order to analyze patients' medical history as well as oral examination and dental radiographs taken and local risk factors are analyzed.

A total of 58 MR-ONJ patient data were consecutively analyzed. Patients characteristics, demographic data, frequency of cancer location, lines of therapy, frequency of oncologic drugs, oral conditions, number, site, and staging of jaw osteonecrosis were reported. Zoledronic acid duration and cumulative dose were evaluated. Previous and concurrent cancer treatments with antineoplastic agents including denosumab and bevacizumab and corticosteroids were also reported. Registered MR-ONJ features (location and number of MR-ONJ lesions, stage, presence of suppuration, and referred oral trigger) were analyzed.

MR-ONJ stage was classified according to the Italian Society of Oral Medicine and Pathology (SIPMO) approach.

## 3. Results

Patients characteristics are summarized in Table 1 including details in relation to gender, frequency of cancer location, frequency of oncologic drugs other than zoledronic acid, frequency of MR-ONJ location, presence/absence of oral trigger, and presence/absence of suppuration among subjects who developed osteonecrosis of the jaws. Female gender was prevalent representing 60% of patients. The most frequent cancer type was breast cancer (65,5%) followed by prostate cancer (27,5%). In the registry, we found 3 patients who were given antiresorptive therapy for metastatic lung cancer (5%) and 1 patient diagnosed with nasopharynx cancer.

Of the 58 patients in the registry, all the patients received corticosteroids; all the patients but two received zoledronic acid, two patients received denosumab as the only antiresorptive treatment for bone metastasis, and 15,5% of the patients received both zoledronic acid and denosumab.

Docetaxel was found to be administered in 65,5% of the patients while in 43% of the patients paclitaxel was the administered taxan, and five patients were treated sequentially with docetaxel and paclitaxel. 24% of the patients were given bevacizumab.

In relation to MR-ONJ features, the most common location of MR-ONJ was the mandible (*n* = 43  [74%]), followed by the upper maxilla (*n* = 10  [18%]). Furthermore 5 cases involving both jaws (9%) were observed. MR-ONJ lesions were reported to be mainly symptomatic due to the presence of suppuration, and localized infections were detected in 64% of cases (9 subjects in stage I [16%] and 28 in stage II [48%]). The presence of a potential oral trigger was recorded in 6 (62%) patients.

According to the SIPMO classification, the most frequent stage of MR-ONJ was stage II (37 subjects), whereas stage I (13 subjects) and stage III (8 subjects) were less common (Table 2).

Mean age was 63,43 years. In relation to the length of exposure, the average duration of antiresorptive therapy until the moment of the diagnosis was 17,58 months (ranging from 2 to 48 months), corresponding to 67,46 mg if expressed as average cumulative dose (Table 3).

Patients were mostly receiving first-line (28%) or second-line (29%) cancer therapy, more patients received third-line chemotherapy (21%), a less percentage of patients who undergo advanced (greater than third) chemotherapy lines was reported, and fourth- and fifth-line therapy were used in 17% and 5% of the patients, respectively (Table 4).

## 4. Discussion

MR-ONJ is a severe adverse drug reaction consisting of progressive bone destruction in the maxillofacial region of patients. MR-ONJ can be caused by several pharmacological agents, mainly antiresorptive (including bisphosphonates (BPs) and receptor activator of nuclear factor kappa-B ligand inhibitors) and antiangiogenic [15, 16].

A major obstacle in elucidating the pathophysiology of this disease is the simultaneous use of multiple therapeutic drugs in cancer patients, which are required to control both tumor growth and its related skeletal complications [17–20]. The study highlighted the role of taxanes (docetaxel and paclitaxel) a second-line monotherapy or combination therapy which is an effective option in the treatment of patients with metastatic breast cancer after failure of prior chemotherapy [21]. Indeed results reported that in the majority of the patients (29%) MR-ONJ occurred while receiving a second-line cancer therapy.

Docetaxel targeting endothelial cells inhibits endothelial cell proliferation and tubule formation in a dose-dependent fashion [9]. Taxanes have remained a cornerstone of breast cancer treatment [21] particularly in triple negative subsets [22].

In this study, we investigated the role of taxanes, in a broader sense of chemotherapeutic agents in the development of this serious side effect. In this case series of 58 patients who were concurrently given taxanes and zoledronic acid/denosumab, MR-ONJ occurred mostly in advanced stages (diffuse osteonecrosis with presence of infection in the 64% of the cases).

MR-ONJ involved the lower jaw more frequently than the upper or both jaws and the presence of a potential oral trigger was identified in the majority of the cases, results in line with previously published data [2].

MR-ONJ stage was classified according to the Italian Society of Oral Medicine and Pathology (SIPMO) approach, based on the extent of bone disease, i.e., whether it is focal or diffuse assessed through CT scan and the clinical findings of presence/absence of suppuration [23–26].

The findings show that patients who are given taxanes and corticosteroids for metastatic cancer while undergoing zoledronic acid or denosumab treatment are prone to developing MR-ONJ.

Nevertheless, the development of MR-ONJ under bone metastasis treatment may be associated solely with the duration of ZA/denosumab treatment. The concurrent administration of docetaxel could have eventually allowed a faster worsening of the clinical manifestation through the exacerbation of soft tissue defects due to chemotherapy drugs.

Moreover during the administration of taxanes, the systematic premedication with antihistamines and corticosteroids before standard infusions is used to prevent infusion adverse reactions. Consequently, all the patients assumed a considerable dose of corticosteroids and these medications are associated with an increased risk for MR-ONJ development [27, 28]. After all in the literature, it has been already hypothesized that combining bisphosphonates with other medications such as antiangiogenic agents may induce MR-ONJ more frequently than using bisphosphonates alone [29].

In a literature review by Mcgowan et al., 11 dental risk factors for MR-ONJ development were reported, among these infections/abscesses, although no statistical analysis of the significance of each of these factors was possible [30].

According to the findings from the present study, there was a higher percentage of cases in advanced stages of osteonecrosis, frequently complicated with infection (48% of the patients with diffuse IIB stage disease).

MR-ONJ symptomatology is characterized by dull and ceaseless pain, in advanced stages, and the exposure of necrotic bone is evident, which is frequently associated with purulent secretions and foetor oris [31].

The presence of suppuration was registered in 64% of the patients suggesting the use of targeted antibiotic therapy due to the opportunistic characteristics of the bone suprainfection [31].

Adequate differential diagnosis of microorganisms in the oral cavity prior to therapeutic attempts is of great importance to prevent the spread of MR-ONJ since pharmacological treatment is considered essential in order to control pain and bone infection and preserve patient quality of life [11, 17].

Various other novel antineoplastic and bone-targeting therapies that can also cause jaw necrosis have recently become available [27].

Agents such as corticosteroids, erythropoietin, angiogenic inhibitors (e.g., thalidomide, sunitinib, bevacizumab, and lenalidomide), and tyrosine kinase inhibitors, which are essentially administered as adjuvants in the treatment of cancer patients, have been shown to increase the risk of MR-ONJ when used concomitantly with bisphosphonates or denosumab [32, 33].

In bone metastatic cancer patients, the introduction and increase of the use in combination with bone antiresorptive therapies of a range of chemotherapies and biological targeted therapies are associated with an increase of treatment-related oral toxicities [34].

Oral injuries such as mucosal inflammation and stomatitis go hand in hand with the lack of patients compliance to oral hygiene that can increase the risk of developing MR-ONJ [35]. In the author's opinion, the combination of zoledronic acid and taxanes, plus corticosteroids, may have some synergy of effect and a particularly detrimental influence on the severity of MR-ONJ. The observation of these patients represents a starting point for reflection to stimulate the attention of the community to look after the patients treated with bone targeted therapies in combination with other agents and maximize their mouth care [36, 37].

### 4.1. Limitations of the Study

The authors consider it worth underlining that in their opinion studies conducted in a osteonecrosis of the jaws treatment center are biased since they do not represent the true frequency of MR-ONJ because this institute serves mainly as a referral center for oncologists and mainly problematic cases are submitted for consultation.

The best source to obtain information on the true relative frequency of MR-ONJ is from the records of a large cancer center.

Information gained from the files of such an institution is invaluable and represents the only large source of data for a case and control study on the relative frequency of MR-ONJ.

## 5. Conclusions

This study evaluated data obtained from patients with metastatic cancer, whose files and treatments were regularly followed up in the osteonecrosis of the jaws treatment centers of the University of Palermo and Messina.

A multicentric case and control study has already been designed for a comparative evaluation of MR-ONJ occurrence matching the case group with a population of cancer patients with current or previous treatment with the same type of medication (zoledronic acid and denosumab) for metastatic bone disease and had not developed MR-ONJ. In the absence of data from a case and control study, no firm conclusion can be drawn about the synergistic effect of combined ZA/denosumab and chemotherapeutic agents in developing MR-ONJ. Anyhow in patients receiving bone targeted therapies in combination with cancer therapies associated with oral complications, intense clinical observation is even more recommended.

## Figures and Tables

**Table 1:** Patients characteristics: gender, frequency of cancer location, frequency of oncologic drugs other than zoledronic acid, frequency of MR-ONJ location, presence/absence of oral trigger, and presence/absence of suppuration among subjects who developed osteonecrosis of the jaws.

	*n* (%)
*Gender*	
Female	36 (62%)
Male	22 (38%)
*Cancer location*	
Breast cancer	38 (65,5%)
Prostate cancer	16 (27,5%)
Lung cancer	3 (5%)
Nasopharynx cancer	1 (2%)
*Other therapies*	
Docetaxel	38 (65,5%)
Paclitaxel	25 (43%)
Bevacizumab	14 (24%)
Denosumab	9 (15,5%)
*ONJ location*	
Mandibular	43 (74%)
Maxilla	10 (18%)
Mandibular/Maxilla	5 (9%)
*Oral trigger*	
Absence	22 (38%)
Presence	36 (62%)
*Suppuration*	
Absence	21 (36%)
Presence	37 (64%)

**Table 2:** MR-ONJ stage.

MR-ONJ stage	*n* (%)
IA	7 (12%)
IIA	6 (10%)
IB	9 (16%)
IIB	28 (48%)
III	8 (14%)

**Table 3:** Patients age and description of zoledronic acid therapy regimen.

	Min	Mediana	Media	Max	SD
Age	43	64	63,43	84	10,24
Cumulative dosage ZA	8	62	67,46	192	37,8
Therapy duration	2	16	17,58	48	10,08

**Table 4:** Lines of cancer therapy.

Line of cancer therapy	*n* (%)
1	16 (28%)
2	17 (29%)
3	12 (21%)
4	10 (17%)
5	3 (5%)

## Data Availability

MR-ONJ cases are usually recorded in a specific dataset, data are fully anonymized, and a patient ID is assigned. Data for this retrospective analysis are obtained from this constantly expanding dataset and are available from the corresponding author on reasonable request.
